# Treatment of tibial bone defects caused by infection: a retrospective comparative study of bone transport using a combined technique of unilateral external fixation over an intramedullary nail versus circular external fixation over an intramedullary nail

**DOI:** 10.1186/s12891-024-07377-2

**Published:** 2024-04-12

**Authors:** Xiayimaierdan Maimaiti, Kai Liu, Aihemaitijiang Yusufu, Zengru Xie

**Affiliations:** Department of Trauma and Microreconstructive Surgery, The First Affiliated Hospital of Xinjiang Medical University, Urumqi, Xinjiang 830054 China

**Keywords:** Bone defect, Bone transport, External fixator, Ilizarov method, Infection

## Abstract

**Background:**

The purpose of the study was to assess and compare the clinical efficacy of bone transport with either circular or unilateral external fixators over an intramedullary nail in the treatment of tibial bone defects caused by infection.

**Methods:**

Between May 2010 and January 2019, clinical and radiographic data were collected and analyzed for patients with bone defects caused by infection. Thirteen patients underwent bone transport using a unilateral external fixator over an intramedullary nail (Group A), while 12 patients were treated with a circular external fixator over an intramedullary nail (Group B). The bone and functional outcomes of both groups were assessed and compared using the Association for the Study and Application of the Method of the Ilizarov criteria, and postoperative complications were evaluated according to the Paley classification.

**Results:**

A total of 25 patients were successfully treated with bone transport using external fixators over an intramedullary nail, with a mean follow-up time of 31.63 ± 5.88 months. There were no significant statistical differences in age, gender, previous surgery per patient, duration of infection, defect size, and follow-up time between Group A and Group B (*P* > 0.05). However, statistically significant differences were observed in operation time (187.13 ± 21.88 min vs. 255.76 ± 36.42 min, *P* = 0.002), intraoperative blood loss (39.26 ± 7.33 mL vs. 53.74 ± 10.69 mL, *P* < 0.001), external fixation time (2.02 ± 0.31 month vs. 2.57 ± 0.38 month, *P* = 0.045), external fixation index (0.27 ± 0.08 month/cm vs. 0.44 ± 0.09 month/cm, *P* = 0.042), and bone union time (8.37 ± 2.30 month vs. 9.07 ± 3.12, *P* = 0.032) between Group A and Group B. The excellent and good rate of bone and functional results were higher in Group A compared to Group B (76.9% vs. 75% and 84.6% vs. 58.3%). Statistically significant differences were observed in functional results (excellent/good/fair/poor, 5/6/2/0 vs. 2/5/4/1, *P* = 0.013) and complication per patient (0.38 vs. 1.16, *P* = 0.012) between Group A and Group B.

**Conclusions:**

Bone transport using a combined technique of external fixators over an intramedullary nail proved to be an effective method in treating tibial bone defects caused by infection. In comparison to circular external fixators, bone transport utilizing a unilateral external fixator over an intramedullary nail resulted in less external fixation time, fewer complications, and better functional outcomes.

## Background

The Ilizarov bone transport technique, which involves bone transport, is a commonly utilized method for treating bone defects resulting from various pathological conditions such as limb discrepancy, direct trauma, chronic osteomyelitis, and resection of bone tumors [1–4]. While it has proven effective in correcting deformities and repairing bones, drawbacks such as pin tract infections, the delayed union of the docking site, and psychological burdens due to long external fixation times still exist [5]. The achievement of satisfactory bone and functional results mainly depends on the mineralization status of the regenerated bone in the distraction area, which is closely related to the chosen treatment strategy [6]. Consequently, long external fixation times and the risks of postoperative complications remain significant challenges for orthopedic surgeons utilizing the Ilizarov bone transport technique.

Previous studies have suggested new techniques to shorten external fixation time, such as multi-level bone transport [6, 7], induced membrane followed by trifocal bone transport [8], and bone transport using external fixation over an internal fixation (plate/intramedullary nail) [9]. Among these methods, bone transport using a combined technique of an external fixator over an intramedullary nail (EFOIN) has been shown to provide stable fixation and significantly reduce the external fixation index and the incidence of complications such as axial deviation [9]. This technique can also stimulate internal reparative osteogenesis by reaming the bone marrow canal during nailing, and the external frame can be removed after the distraction phase to facilitate satisfactory limb functional recovery and allow patients to return to normal life. Despite the acknowledged advantages of bone transport using EFOIN, uncertainties persist regarding the specific surgical procedures and clinical outcomes associated with different types of external fixation within this combined technique. Therefore, this study aims to assess and compare the clinical efficacy of bone transport using either circular or unilateral external fixators over an intramedullary nail for the treatment of tibial bone defects caused by infection.

## Materials and methods

The clinical data and radiography of all patients were retrospectively evaluated from May 2010 and January 2019, after receiving written informed consent from participants and approval from the Ethics Committee of our hospital. Inclusion criteria are as follows: tibial bone defect caused by infection; sinus tract and positive intraoperative culture supporting a deep bony infection of the affected limb; treated by bone transport using EFOIN. Exclusion criteria were incomplete medical records, poor compliance, or a follow-up time of fewer than twenty months [10].

Inflammatory markers were recorded and retrospectively analyzed, such as C-reactive protein, white blood cell, procalcitonin, and erythrocyte sedimentation rate. Cierny and Mader’s (CM) classification was used to evaluate the degree of bone infection. The patient received antibiotics treatment for 2 weeks before the surgery, based on the results of bacterial culture and drug sensitivity test.

### Patients’ data

The cohort included 25 patients (20 males and 5 females), with a mean age of 41.13 ± 7.4 years (Table 1). Thirteen patients were treated by bone transport using a unilateral external fixator over an intramedullary nail (Group A) and 12 patients with a circular external fixator over an intramedullary nail (Group B). All patients had sinus tracts of the affected limb and positive results of the culture test. Pathologic mechanisms of bone defects included 21 cases (84%) with post-traumatic osteomyelitis, and 4 cases (16%) with chronic osteomyelitis. The mean duration of infection was 29.8 ± 5.73 months, and patients had undergone a mean of 2.9 previous surgical procedures. Based on CM classification, 15 patients (60%) were type III, and 10 patients (40%) were type IV. Bacteria were identified in all cases (100%), with *Staphylococcus aureus* being the most common (76%).

**Table 1:** Baseline data of patients

Variables	Group A (*n* = 13)	Group B (*n* = 12)	t / *χ*^2^	*P* value
Male	11	9	0.740	0.685
Age (years)	42.5 ± 7.39	40.84 ± 6.43	1.045	0.374
Initial injury mechanism (traffic accident/crushing/falling)	8/3/2	8/2/2	0.201	0.851
Initial treatment (plate/intramedullary nailing/ external fixation)	4/6/3	5/3/4	0.470	0.506
Receiving free flap or skin graft (%)	6	5	1.862	0.085
Positive results of bacteria culture (S. aureus/Staphylococcus epidermidis/ E. coli)	7/4/2	6/3/3	0.528	0.634
Previous surgery per patient	3	2.8	0.451	0.503
Duration of infection (month)	16.62 ± 5.04	15.54 ± 3.15	0.647	0.524
DS (cm)	7.3 ± 0.61	6.78 ± 0.91	1.682	0.106
Operation time (minute)	187.13 ± 21.88	255.76 ± 36.42	2.994	0.002
Intraoperative blood loss (mL)	39.26 ± 7.33	53.74 ± 10.69	3.961	< 0.001
BUT (month)	8.37 ± 2.30	9.07 ± 3.12	2.890	0.032
EFT (month)	2.02 ± 0.31	2.57 ± 0.38	2.634	0.045
EFI (month/cm)	0.27 ± 0.08	0.44 ± 0.09	2.771	0.042
Complication (per patient)	5 (0.38)	14 (1.16)	2.315	0.012
Follow-up time (months)	30.88 ± 5.77	34.82 ± 3.65	0.681	0.055

BUT, bone union time; DS, defect size; Escherichia coli, E. coli; EFT, external fixation time; EFI, external fixation index; Staphylococcus aureus, S. aureus

### Surgical procedure

The surgical procedure involved removing necrotic bone and soft tissue until the “paprika sign” was seen on the bone, followed by flushing the area with 0.9% saline under low pressure. Surgeons replaced their gloves and instruments before making a midline incision of the anterior knee ligament, layer by layer to the tibial plateau. An intramedullary guide wire (3.5 mm Steinmann pin) was then inserted at an insertion point below the tibial plateau and drilled and reamed in the direction of the tibial medullary canal. An appropriate length of intramedullary nail was inserted and secured, followed by a minimally invasive osteotomy procedure utilizing a sharp osteotome. Particular attention was paid to ensuring the osteotome only penetrated the tibial cortex without reaching too deeply into the bone, thereby mitigating the risk of damaging the intramedullary blood supply and the integrity of the intramedullary nail. The circular external fixator was used in Group A, while a unilateral external fixator was applied in Group B. After assembling the clamps and rods of the external fixator, a tension-free direct suture was performed. The external fixator was removed at the end of the distraction phase, but the internal fixator remained in place until the consolidation phase was complete.

### Postoperative management

The distraction phase began at 5–7 days postoperatively in both groups, with a rate of 1 mm/day until the docking site was connected. Patients were encouraged to start active and passive knee range of motion exercises, without weight-bearing, as soon as possible. Weight-bearing walking was allowed during the consolidation phase. Patients were given instructions on pin tract care to avoid infection. The dynamic compression-distraction technique (5 - day compression and 5 - day distraction at a rate of 0.5 mm/12 h for 10 days) was performed to prevent delayed union before removing the external fixator. The external fixator was removed upon verification of the continuous absence of bending deformity or delayed union at the docking site over a consecutive 2-week period after the dynamic compression-distraction technique. A knee hinge brace was used to protect weight-bearing activity for approximately 3 weeks. Radiography of the affected limb was examined at 1, 3, 6, 9, 12, 18, and 24 postoperative months.

### Data collection and outcome evaluation

The demographic data, length of bone defect, bone union time (BUT), external fixation time (EFT), external fixation index (EFI), follow-up time, and complications in the two groups were documented and compared. Bone and functional outcomes were evaluated using the ASAMI criteria, and complications were recorded according to Paley’s classification (minor was defined as not requiring additional surgery, and major was defined as either resolved with additional surgery or remaining unresolved).

### Statistical analysis

Data were analyzed by the SPSS 23.0 software package (Chicago, IL, USA). The Shapiro-Wilk test was used to assess data normality. Continuous variables were expressed as the mean and standard deviation, and the independent samples t-test or Mann-Whitney U test was used for comparison between the two groups. Categorical variables were analyzed by the chi-square test. Statistical significance was *P* < 0.05.

## Results

A total of 25 patients were successfully treated with bone transport using EFOIN, with a mean follow-up time of 31.63 ± 5.88 months. Bone infection was eradicated in all cases without postoperative recurrence of infection, and the mean length of the bone defect was 6.92 ± 0.72 cm. There were no significant statistical differences in age, gender, previous surgery per patient, duration of infection, DS, and follow-up time between Group A and Group B (Table 1, *P* > 0.05). However, statistically significant differences were observed in operation time (187.13 ± 21.88 min vs. 255.76 ± 36.42 min, *P* = 0.002), intraoperative blood loss (39.26 ± 7.33 mL vs. 53.74 ± 10.69 mL, *P* < 0.001), EFT (2.02 ± 0.31 month vs. 2.57 ± 0.38 month, *P* = 0.045), EFI (0.27 ± 0.08 month/cm vs. 0.44 ± 0.09 month/cm, *P* = 0.042), and BUT (8.37 ± 2.30 month vs. 9.07 ± 3.12, *P* = 0.032) between Group A and Group B.

**Fig. 1:**
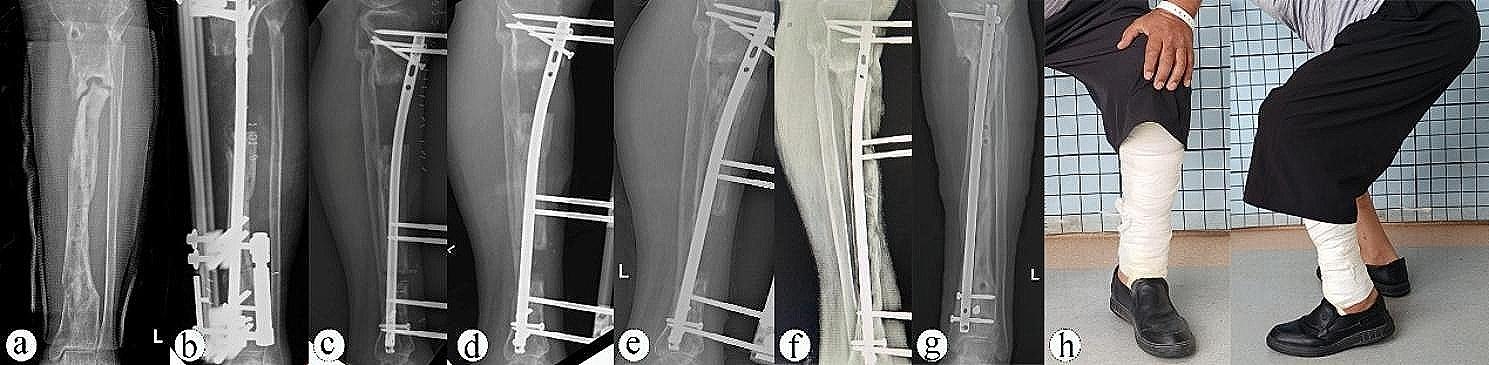
53-year-old male with a left tibial bone defect caused by post-traumatic osteomyelitis was treated by bone transport using EFOIN. **a**) X-ray showed a nonunion of the left tibia before reconstructive surgery. **b**, **c**, **d**) After radical debridement, the bone defect was approximately 7.3 cm and treated by bone transport using a unilateral external fixator over an intramedullary nail. **e**) Callus was observed in the distraction area without axial deviation at 7 postoperative weeks. **f**) The transport bone segment reached the docking site at 11 postoperative weeks. **g**, **h**) Satisfactory bone healing and function recovery of the left lower limb was achieved at 9 postoperative months

**Fig. 2:**
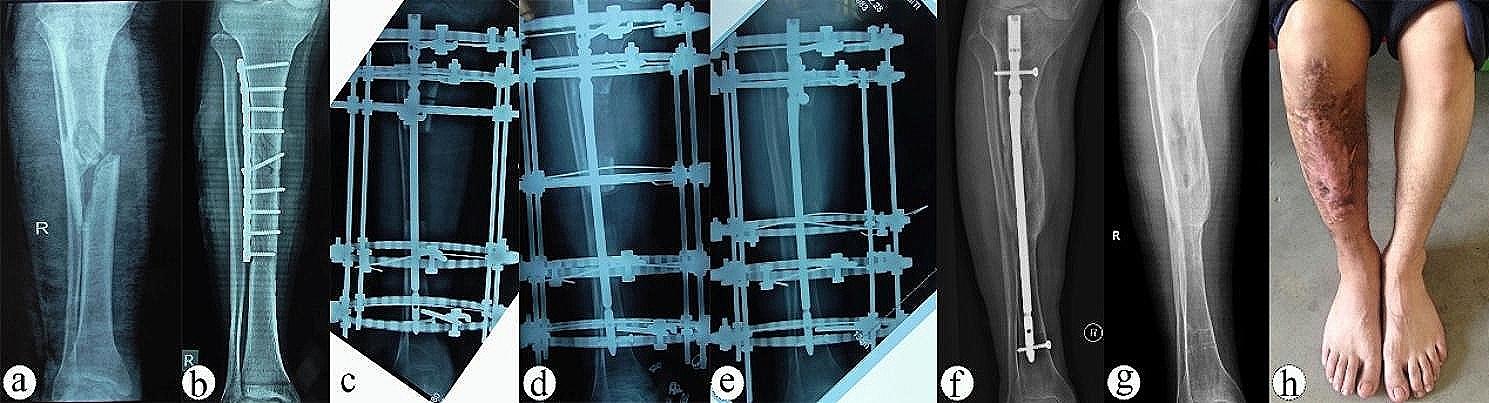
32-year-old male with a right tibial bone defect caused by post-traumatic osteomyelitis was treated by bone transport using EFOIN. **a**, **b**) X-ray showed that there was a nonunion of the right tibia after internal fixation. **c**) The bone defect was approximately 9.8 cm after radical debridement and treated by bone transport using a circular external fixator over an intramedullary nail. **d**, **e**) The transport bone segment reached the docking site at 15 postoperative weeks, and the regenerated callus was good without axial deviation. **f**–**h**) Satisfactory bone healing and function recovery of the right lower limb was achieved at 12 postoperative months

Based on the ASAMI criteria, the excellent and good rates of bone and functional results were higher in Group A compared to Group B (76.9% vs. 75% and 84.6% vs. 58.3%). Statistically significant differences were observed in functional results (excellent/good/fair/poor, 5/6/2/0 vs. 2/5/4/1, *P* = 0.013) and complication per patient (0.38 vs. 1.16, *P* = 0.012) between Group A and Group B (Table 2). Pin tract infection occurred in 9 patients (3 patients in Group A and 6 patients in Group B), which responded effectively to pin tract care and oral antibiotics. Radiating foot pain occurred in 7 patients (2 patients in Group A and 5 patients in Group B). Two patients with adjacent joint stiffness in Group B improved after physiotherapy. One patient in Group B had a limb length discrepancy of about 1 cm. There were no instances of delayed union, nonunion, or re-fracture in either group. Complications in the two groups were classified as minor according to Paley’s classification and were successfully managed without postoperative sequelae. Typical cases treated using the combined EFOIN technique are presented in Figs. 1 and 2.

**Table 2:** Outcomes of ASAMI scores in two groups

ASAMI	Location	Excellent	Good	Fair	Poor	Failure
Bone grade	Group A	3	7	3	0	0
	Group B	4	5	3	0	0
Function grade^*^	Group A	5	6	2	0	0
	Group B	2	5	4	1	0

*P < 0.05

Bone results

Excellent: Union, no infection, deformity < 7°, limb length discrepancy (LLD) < 2.5 cm

Good: Union plus any two of the following: the absence of infection, deformity < 7°, LLD < 2.5 cm.

Fair: Union plus any one of the following: the absence of infection, deformity < 7°, LLD < 2.5 cm.

Poor: Nonunion/refracture/union plus infection plus deformity > 7° plus LLD > 2.5 cm

Functional results

Excellent: Active, no limp, minimum stiffness (loss of < 15°knee extension or < 15°ankle dorsiflexion) no reflex sympathetic dystrophy (RSD), insignificant pain

Good: Active, with one or two of the following: limb, stiffness, RSD, significant pain

Fair: Active, with three or all of the following: limb, stiffness, RSD, significant pain

Poor: Inactive (unemployment or inability to return to daily activities because of injury)

Failure: Amputation

## Discussion

The Ilizarov bone transport technique has been proven to be an effective method for treating tibial bone defects caused by fracture-related infections [1, 3, 11]. The combined technique of EFOIN proposed in previous studies allows for effective control of the alignment of the transported bone segment via an intramedullary nail, thus avoiding axial deviation [12]. Furthermore, this technique can greatly shorten the EFT and EFI by removing the external fixator after the distraction phase, and further reduce the occurrence of external-fixation-related complications [9]. In this cohort, all 25 patients (100%) were successfully treated with bone transport using EFOIN, with a minor complication rate of 0.76. Therefore, bone transport using a combined technique of EFOIN offers significant advantages in restoring lower limb alignment and reducing postoperative complications.

The Ilizarov bone transport technique using a circular external fixator has some limitations, such as long treatment time, cumbersome appearance, and high incidence of postoperative complications [5]. Although the application of unilateral external fixators simplifies surgical procedures and improves patient compliance, this technique still faces high EFT and EFI. In recent years, orthopedic researchers have proposed bone transport using EFOIN, which significantly reduces EFT and EFI, including external fixator combined with intramedullary nails [9], and plate-assisted bone segment transport [13]. Guo et al. [14] reported a comparative study of tibial lengthening over an intramedullary nail versus the conventional Ilizarov method and suggested that tibial lengthening over an intramedullary nail conferred advantages in reducing EFT with a lower complication rate. Farsetti et al. [15] presented 28 patients with lower limb discrepancy treated by limb lengthening over an intramedullary nail and considered that this method could reduce EFT, prevent the occurrence of axial deformities, and fractures of regenerated bone. In this study, bone transport utilizing either a circular or unilateral external fixator through the method of EFOIN demonstrated distinct advantages in facilitating bone union (100%) and minimizing both EFT and EFI. However, intraoperative blood loss, operation time, EFT, EFI, and BUT in Group B were less than those of Group A (*P* < 0.05). We posit that the simpler surgical techniques associated with unilateral external fixators contribute to decreased intraoperative periosteal damage, thus fostering enhanced bone regeneration within the distraction zone. Additionally, the implementation of minimally invasive osteotomy plays a crucial role in bone transport surgery by mitigating intraoperative blood loss.

Intramedullary nailing is considered the gold standard for managing long bone fractures in the lower limbs, providing satisfactory axial stability, stiffness, and minimal soft tissue injury [16]. In bone transport, the addition of intramedullary nailing can keep the distracted bone segment stable and minimize EFT and loss of axial alignment. However, some scholars have reported that bending deformities of the distraction area may occur at the proximal tibial osteotomy site when treating distal tibial bone defects using EFOIN [13, 17, 18]. Hence, a plate-assisted bone transport technique was developed. Oh et al. [13] presented a total of 10 patients with infected post-traumatic segmental tibial defects effectively managed by distraction osteogenesis with a locking plate. Lu et al. [19] reported a series of 12 patients with segmental tibial defects successfully treated by a combined bone transport technique of circular external fixation and locking plate application. In our experience, the application of intramedullary nailing can guide the distracted bone segment and provide axial stability during bone transport, preventing the occurrence of axial deviation and bending deformity of the distraction area. The inserted screws of assisted locking plate may harm the periosteal blood supply and leave a high incidence of stress shielding, which may result in pathological bone resorption of the distraction area. Besides, reaming during the use of intramedullary nails can also act as an internal bone grafting to promote bone regeneration in the distraction area. Therefore, although bone transport using a plate may reduce the risk of bending deformities in the distraction area, intramedullary nailing can be a better choice.

Previous studies have reported pin tract infection as the most common complication in external fixation treatment. Additionally, there is a high risk of axial deviation and transport gap bending deformity with bone transport using a unilateral external fixator [3, 11]. In this study, the most common complications were pin tract infections (Group A vs. Group B, 3 cases vs. 6 cases), followed by radiating foot pain (Group A vs. Group B, 2 cases vs. 5 cases). However, neither axial deviation nor transport gap bending deformity was observed. The complication ratio (per patient) in Group A was lower than that of Group B (*P* < 0.05). We consider that more screws or pins are present when using a circular fixator, which increases the risk of pin tract infection. The distraction procedure during the distraction phase of circular external fixation is more complex than that of unilateral external fixation, which may leave a higher risk of irritating peripheral nerves during insertion and further lead to radiating foot pain. This further complicates rehabilitation for patients, as they may experience difficulty performing exercises to improve mobility in adjacent joints, leading to joint stiffness. Therefore, bone transport with a unilateral external fixator over an intramedullary nail can result in lower complication rates and better functional results.

Despite the satisfactory outcomes observed, there is a significant risk of infection spreading to the medullary canal when using intramedullary nails [20]. Although all cases in this study successfully eradicated the infection without recurrence, infected lesions often present difficulties due to previous surgeries, poor soft tissue coverage and circulation. As such, it is crucial to utilize sensitive systemic antibiotics, perform radical debridement, and implement meticulous postoperative management. Patients should also be carefully instructed on pin tract care to prevent pin tract loosening or the spread of infection. Besides, there is an increasing risk of delayed union or nonunion of the docking site as the length of the bone defect size increases. In our cohort, the dynamic compression-distraction technique (5 - day compression and 5 - day distraction at a rate of 0.5 mm/12 h for 10 days) was performed to prevent delayed union before removing the external fixator. However, when a delayed union or nonunion occurs, autologous bone grafting at the docking site was recommended to perform to promote bone healing.

This study had several limitations. Firstly, the absence of a standardized algorithm for managing tibial bone defects caused by infection may have affected the correlation between various treatment methods. Furthermore, interpretations of bone and functional outcomes should be approached with caution, due to the retrospective nature and small sample size of this study. Therefore, it is crucial to conduct a large-scale, multi-center, and prospective study to accurately evaluate the clinical efficacy of bone transport using EFOIN.

## Conclusion

Bone transport using a combined technique of EFOIN proved to be an effective method in treating tibial bone defects caused by infection, as it significantly reduced EFT, EFI, and the incidence of complications. Dynamic compression-distraction technique was a practical tool to prevent the risk of docking site nonunion. In comparison to circular external fixators, bone transport utilizing a unilateral external fixator over an intramedullary nail resulted in less external fixation time, fewer complications, and better functional outcomes.

## Data Availability

The data sets generated and analyzed during the current study were not publicly available due to restrictions on ethical approvals involving patient data and anonymity but can be obtained from the corresponding author on reasonable request.
